# Do no harm: managing nausea and vomiting in GLP-1 based obesity therapies

**DOI:** 10.3389/fendo.2026.1788698

**Published:** 2026-03-03

**Authors:** Abdulhameed Alhazmi, Carel W. le Roux

**Affiliations:** 1 Internal Medicine, Jazan University Hospital, Jazan, Saudi Arabia; 2 Diabetes Complications Research Centre, University College Dublin, Dublin, Ireland; 3 Diabetes Research Centre, Ulster University, Belfast, United Kingdom

**Keywords:** GLP 1, nausea & vomiting, obesity, semaglutide, side effect, tirzepatide

## Introduction

The advent of new obesity medications has transformed the care of the disease of obesity, reframing weight loss from an aspirational goal to a realistic therapeutic outcome. Disease modifying agents capable of controlling obesity with meaningful metabolic improvement have reshaped expectations for both clinicians and patients (1). However, as efficacy has advanced, tolerability has emerged as a central clinical challenge for long term compliance with these medications (2). Adverse effects: particularly gastrointestinal can limit the benefits of treatment, especially if treatment is discontinued (3). The clinical challenge is therefore to manage treatment burden in a way that prevents early discontinuation, because only sustained, long-term therapy can optimise metabolic, cardiovascular, and weight related benefits.

Nausea and vomiting remain among the most frequent and disruptive adverse effects across Glucagon-like peptide (GLP-1)-based therapies (4). As the treatment landscape expands beyond GLP-1 only receptor agonists to include multi-agonist and oral agents, these symptoms continue to influence initiation, titration, and persistence with therapy. Although gastrointestinal intolerance is often most pronounced during early dose escalation and may attenuate over time, substantial inter-individual variability persists, highlighting the gap between therapeutic efficacy and real-world acceptability (4).

In this opinion article, we examine the mechanisms underlying nausea and vomiting in semaglutide, CagriSema, survodutide, tirzepatide, retatrutide, and orforglipron and situate them within obesity clinical practice (5–14). Importantly, approximately 60–70% of patients can titrate to the full therapeutic dose and with minimal difficulty, experiencing little to no gastrointestinal adverse effects (8, 11, 14). However, the remaining 30–40% may struggle during the escalation phase, developing nausea, vomiting, or treatment-limiting intolerance (14). Thus, the existing titration schedules used in phase 3 clinical trials can be appropriate for the majority of patients, but more attention should be given to the minority of patients who do have substantial side effects. The challenge remains that prior to treatment initiation it remains virtually impossible to predict who will have side effects (3).

We also propose practical strategies to mitigate gastrointestinal intolerance; particularly nausea and vomiting to support treatment continuity. The goal is to help patients remain on an effective therapy long enough to achieve sustained metabolic and cardiovascular benefit without unnecessary interruption. A helpful framework comes from insulin titration. With insulin, mild hypoglycaemia signals that dose escalation is progressing faster than the body can handle, prompting a pause or small adjustment. Severe hypoglycaemia indicates clearly that the insulin dose is too high requiring a more substantial correction. Titration schedules in phase 3 trials of long acting insulin analogues incorporated individualization to allow the clinician to increase the dose to achieve optimal benefit without causing hypoglycaemia (15). Similarly, with GLP-1 therapy, nausea functions like mild hypoglycaemia, which can be seen as an early warning to slow or temporarily pause titration. Vomiting parallels severe hypoglycaemia, indicating intolerance significant enough to stepping back to the last well tolerated level. This parallel reinforces that symptoms are not failure, but physiological feedback guiding safer, more individualized titration.

## The oral obesity medications

The oral GLP-1 based agents approved for obesity treatment deliver substantial weight loss. Oral semaglutide 25–50 mg produces the largest average reduction, followed by orforglipron closely behind (5–7). The main practical difference lies in how patients take them: both semaglutide tablets require a fasting routine and specific timing around meals, while orforglipron can be taken more flexibly, which may improve convenience. However, oral semaglutide remains an acylated peptide with very low bioavailability (<1%) and highly variable day-to-day absorption, resulting in larger fluctuating plasma concentration compared with subcutaneous dosing. This variability may contribute to a higher burden of gastrointestinal adverse events. In contrast, orforglipron is a small molecule agent with more predictable absorption and lower variability both within and between individuals.

Nausea and vomiting increase with higher semaglutide exposure, with the 50 mg oral dose showing the greatest frequency, especially during dose escalation. Orforglipron shows typical GLP-1-related gastrointestinal effects and discontinuation due to gastrointestinal symptoms appears similar compared to oral semaglutide. This suggests that convenience of dosing does not fully offset tolerability challenges (5–7). **Table 1** offer a comparison between the oral based GLP-1 agents.

**Table 1:** Oral based GLP-1 agents.

Feature	Oral semaglutide 25mg (5)	Oral semaglutide 50mg (6)	Orforglipron 36mg (7)
Type	Peptide GLP-1RA + SNAC	Peptide GLP-1RA + SNAC (higher exposure)	Small-molecule GLP-1RA (non-peptide)
Dosing routine	Daily, fasting dose → strict 30-min wait	Same as the 25 mg	Daily; no strict fasting
Mean weight loss	~13.6% by week 64	~15.1% by week 68	~11.2% by week 36 (sustained to week 72)
Nausea	~47%	~52%	~33.7%
Vomiting	~31%	~24%	~24%

GLP-1RA, glucagon-like peptide-1 receptor agonist; SNAC, sodium N-(8-[2-hydroxybenzoyl]amino) caprylate.

## The injectable obesity medications

Semaglutide 2.4 mg, a selective GLP-1 receptor agonist, produces robust and durable weight loss of approximately 15–16% over 68 weeks (8, 10). Nausea occurs in roughly half of treated patients and vomiting in around one quarter, typically clustering during dose escalation and declining with continued treatment (4). Semaglutide 7.2 mg extends this same biological mechanism to a higher exposure range and achieves greater weight reduction, approaching low twenties percentage range depending on the estimand used (9). This gain in efficacy is accompanied by a higher burden of gastrointestinal symptoms, again dominated by nausea (43.7%) and vomiting (24.8%), but largely remaining mild to moderate and transient, with many patients not having any side effects. CagriSema, combining semaglutide with the amylin analogue cagrilintide, enhances weight loss beyond semaglutide alone but increases the frequency of gastrointestinal adverse events, particularly nausea (55%) and vomiting (24.7%), again mainly early in treatment (14). Again many patients using CagriSema have no side effects despite the rapid dose escalation used in phase 3 trials.

Tirzepatide, a dual gastric inhibitory polypeptide (GIP)/GLP-1 receptor agonist, delivers even greater weight loss, exceeding 20% at the highest dose in pivotal trials (4, 11). Its gastrointestinal profile remains consistent with GLP-1–based therapy, with nausea (33%) and vomiting (12%) among the most common adverse events and clear dose dependency. Although many patients don’t have side effects or tolerate these side effects with appropriate titration, gastrointestinal symptoms remain the main driver of treatment discontinuation in a minority of patients (4).

Retatrutide, a triple agonist targeting GIP, GLP-1, and glucagon receptors, represents a further step in efficacy, with 24.2% weight loss achieved within 48 weeks in phase 2 studies (13) and 29% weight loss achieved over 68 weeks in a phase 3 study (16). This enhanced metabolic effect is again coupled with higher rates of gastrointestinal adverse events, particularly nausea (43.2%) and vomiting (20.9%) during dose escalation, reinforcing that multi-receptor engagement does not reduce tolerability challenges.

Survodutide, an oxyntomodulin analogue, achieved clinically meaningful weight loss (12) in phase 2 trials, but nausea affected more than half of participants at higher doses, and vomiting occurred in approximately one quarter, predominantly during the escalation phase, although many patients did not have side effects (12). An illustrated details of the injectable therapies are shown in **Table 2**.

**Table 2:** Injectable based GLP-1 agents.

Feature	Semaglutide 2.4 mg (4, 8)	Semaglutide 7.2 mg (9)	CagriSema 2.4 mg (14)	Tirzepatide 15mg (4, 11)	Retatrutide 12 mg (13, 16)	Survodutide 4.8 mg (12)
Type	GLP-1 receptor agonist	GLP-1 receptor agonist	Amylin analogue + GLP-1 receptor agonist	Dual GIP/GLP-1 receptor agonist	Triple GIP/GLP-1/glucagon receptor agonist	Dual GLP-1/glucagon receptor agonist
Dosing routine	Once-weekly injection	Once-weekly injection	Once-weekly injection	Once-weekly injection	Once-weekly injection	Once-weekly injection
~29% by 68 weeks	~16.0% by 68 weeks	~18.7–20.7% by 72 weeks	~20% by 68 weeks	~21% by 72 weeks	~29% by 68 weeks	~15% by 46 weeks
Nausea	~58%	43.7%	~55%	~30–33%	Up to 43%	~56%
Vomiting	~27%	24.8%	~24.7%	~10–12%	Up to 20.9%	~27%

GLP-1RA, glucagon-like peptide-1 receptor agonist; GIP, glucose-dependent insulinotropic polypeptide; GCGR, glucagon receptor; GIP/GLP-1RA, dual glucose-dependent insulinotropic polypeptide and glucagon-like peptide-1 receptor agonist; GLP-1/GCGR, dual glucagon-like peptide-1 and glucagon receptor agonist; GIP/GLP-1/GCGR, triple glucose-dependent insulinotropic polypeptide, glucagon-like peptide-1, and glucagon receptor agonist; AMY, amylin analogue.

## Pathophysiology of GLP-1 therapy induced nausea and vomiting

GLP-1 receptor agonists cause nausea and vomiting mainly by binding GLP-1 receptors in the dorsal vagal complex (DVC), a specific vomiting and nausea control center in the brainstem. This region includes the nucleus tractus solitarius and the area postrema, which together receive and integrate signals from the gut and the bloodstream. Moreover, when GLP-1–based medications are administered, they also stimulate peripheral GLP-1 receptors on vagal nerve fibers that carry sensory information from the stomach and intestines to the brainstem. This amplifies gut-to-brain signaling and increases the brain’s sensitivity to gastrointestinal sensations (17).

The blood–brain barrier around the area postrema, is more porous thus allowing it to act as a chemosensory trigger zone for nausea and vomiting (18). Thus GLP-1 receptor agonists can directly activate the area postrerma partly explaining the emetic effects observed with GLP-1 therapy (18). In parallel, GLP-1 receptor activation in the gastrointestinal tract slows gastric emptying and alters gastric motility, leading to gastric distension and increased vagal afferent firing. These peripheral effects may further reinforce central emetic signaling, particularly during treatment initiation and dose escalation, when gastric accommodation mechanisms may be less adapted (19). With continued treatment, adaptive changes within these brainstem circuits likely explain why nausea and vomiting often diminish over time.

Unfortunately, beyond the specific GLP-1 pathways, multi-agonist therapies do not bypass the gastrointestinal adverse events despite broader receptor targeting. Although GIP receptor activation may exert anti-emetic effects through brainstem modulation, these effects appear insufficient to completely counterbalance GLP-1 mediated emesis during early treatment and dose escalation (20). This likely explains why dual GLP-1/GIP agonists, such as tirzepatide, continue to show dose dependent nausea, particularly during titration, despite modest improvements in tolerability compared with GLP-1 monotherapy (20).

In contrast, glucagon receptor activation is independently associated with nausea and vomiting through both central and peripheral mechanisms, including effects on gastric motility and autonomic signaling. Consequently, dual GLP-1/glucagon agonists survodutide, and triple GLP-1/GIP/glucagon agonists retatrutide are not spared gastrointestinal adverse events, as glucagon related pro-emetic signaling may offset any potential tolerability benefits from GIP agonism (12, 13, 21).

The contribution of amylin-based therapies to gastrointestinal adverse events remains less clear. Available clinical data indicate that adding cagrilintide to semaglutide does not substantially increase nausea or vomiting compared with semaglutide alone (8, 9, 14). This aligns with the pharmacology of cagrilintide as a dual amylin and calcitonin receptor agonist (DACRA), in which central satiety effects may predominate over peripheral emetic pathways, although mechanistic data remain limited (14).

## Management of GLP-1 induced nausea and vomiting

The cornerstone of preventing nausea and vomiting with GLP-1–based therapy is understanding that gastrointestinal adverse effects are primarily dose related rather than drug specific. Communicating this principle to patients early can shape expectations, reduce anxiety, and improve overall tolerability. Patients should be informed that gastrointestinal symptoms are dose dependent, common during treatment initiation and escalation, usually transient, influenced by the starting dose, and speed of titration (3). Clear communication is essential to reassure patients that the presence or severity of nausea does not predict superior weight loss or metabolic outcomes and may, in fact, undermine long term adherence (22). Framing side effects as a modifiable consequence of dosing strategy allows both patients and clinicians to view dose management as the most effective tool for minimizing intolerance.

Adjunctive dietary and behavioral measures may aid during treatment initiation and dose escalation. GLP-1 analogues delay gastric emptying (1), and practices that facilitate gastric accommodation, such as eating slowly, avoiding high fat or large volume meals, and stopping meals early at the first sensation of fullness may help reduce nausea (3). Patients may also experience symptom relief by avoiding lying flat immediately after meals and maintaining hydration through small, frequent sips rather than large fluid volumes (3). However, these measures are substantially less effective if dose titration is not actively and thoughtfully managed.

When initiating GLP-1based therapy, clinicians should adhere to the principle of *do no harm*, particularly during the early phases of treatment when gastrointestinal adverse effects are most likely to emerge (3). Evidence from a randomized study comparing standard versus gradual, flexible semaglutide titration demonstrates that slower escalation significantly reduces nausea without compromising efficacy (22). In this study, nausea occurred in 64.2% of patients receiving standard titration compared with 45.1% in the flexible titration group, with a markedly shorter duration of symptoms (6.3 vs 2.9 days). Vomiting was less frequent overall and occurred at similar rates between groups, suggesting it reflects transient peak intolerance rather than cumulative exposure. Importantly, treatment discontinuation due to gastrointestinal adverse events was substantially higher with standard titration (19%) than with flexible titration (2%), and most patients who discontinued were able to successfully restart therapy using a slower escalation approach (22).

Potentially the easiest way to prevent nausea and vomiting is by starting at a low dose (23). Depending on the available device dispensing the medication, this can be lower than the recommended labelled dose when feasible. Again depending on the device, it may be advantageous to advance slowly, with titration intervals extended if symptoms of mild nausea emerge (3, 22). The Common Terminology Criteria for Adverse Events (CTCAE) can be used to grade nausea (24). Grade 1 is characterized by loss of appetite without a meaningful change in eating habits. Grade 2 refers to a reduction in oral intake that does not result in significant weight loss, dehydration, or malnutrition, and Grade 3 represents nausea leading to inadequate oral caloric or fluid intake, where nutritional support such as tube feeding or total parenteral nutrition, or hospitalization, may be indicated. Dose escalation could proceed cautiously in the presence of grade 1 nausea, but continuation of the same dose until the side effects subside may be sensible to reduce the risk of Grade 2 nausea. Escalation of doses should be paused once grade 2 nausea or vomiting develops, as continued dose increases at this stage increases the risk of intolerance and discontinuation. Grade 3 nausea requires a substantial dose de-escalation.

In most cases of Grade 1 or 2 nausea, maintaining the current dose for one to two additional dosing intervals is sufficient to allow symptom resolution. In the case of vomiting, dose escalation should not be resumed until vomiting have fully resolved or nausea returned to Grade 1. If Grade 2 nausea persists despite pausing escalation, or if vomiting occurs, the dose should be reduced to the last well tolerated dose rather than discontinued. Once symptoms resolve, patients may be cautiously rechallenged using the previously tolerated dose, with slower subsequent escalation or extended titration intervals.

Importantly, treatment should be individualized, with no obligation to reach a predefined target or maximum dose. If symptoms recur despite repeated pauses or dose reductions, the patient’s maximum tolerated dose should be accepted as the maintenance dose or switching to a different agent. It is worth emphasizing that the gradual approach used did not reduce weight loss or compromise glycemic efficacy, reinforcing that tolerability, not symptom intensity, should guide dose advancement (22).

A patient centered approach of “starting low and going slow” prioritizes safety and adherence, building on the principle of doing no harm. This approach is more likely to support sustained treatment engagement and durable metabolic benefit.

## References

[1] Son JW le Roux CW Blüher M Nauck MA Lim S . Novel GLP-1-based medications for type 2 diabetes and obesity. Endocr Rev. (2025) bnaf036. doi: 10.1210/endrev/bnaf036, PMID: 41054801

[2] Almohaileb F le Roux C Crotty M . Why do patients with obesity discontinue glucagon-like peptide 1 analogues? Diabetes Obes Metab. (2025) 27:5342–5. doi: 10.1111/dom.16531, PMID: 40521761 PMC12326882

[3] Wharton S Davies M Dicker D Lingvay I Mosenzon O Rubino DM . Managing the gastrointestinal side effects of GLP-1 receptor agonists in obesity: recommendations for clinical practice. Postgrad Med. (2022) 134:14–9. doi: 10.1080/00325481.2021.2002616, PMID: 34775881

[4] Kunutsor SK Seidu S . Safety and tolerability of glucagon-like peptide-1 receptor agonists: A state-of-the-art narrative review. Drugs. (2025) 86:11-36. doi: 10.1007/s40265-025-02263-0, PMID: 41351656

[5] Wharton S Lingvay I Bogdanski P Duque do Vale R Jacob S Karlsson T . Oral Semaglutide at a Dose of 25 mg in Adults with Overweight or Obesity. N Engl J Med. (2025) 393:1077–87. doi: 10.1056/NEJMoa2500969, PMID: 40934115

[6] Knop FK Aroda VR do Vale RD Holst-Hansen T Laursen PN Rosenstock J . Oral semaglutide 50 mg taken once per day in adults with overweight or obesity (OASIS 1): a randomised, double-blind, placebo-controlled, phase 3 trial. Lancet. (2023) 402:705–19. doi: 10.1016/S0140-6736(23)01185-6, PMID: 37385278

[7] Wharton S Aronne LJ Stefanski A Alfaris NF Ciudin A Yokote K . Orforglipron, an oral small-molecule GLP-1 receptor agonist for obesity treatment. N Engl J Med. (2025) 393:1796–806. doi: 10.1056/NEJMoa2511774, PMID: 40960239

[8] Wilding JPH Batterham RL Calanna S Davies M Van Gaal LF Lingvay I . Once-weekly semaglutide in adults with overweight or obesity. N Engl J Med. (2021) 384:989–1002. doi: 10.1056/NEJMoa2032183, PMID: 33567185

[9] Wharton S Freitas P Hjelmesæth J Kabisch M Kandler K Lingvay I . Once-weekly semaglutide 7·2 mg in adults with obesity (STEP UP): a randomised, controlled, phase 3b trial. Lancet Diabetes Endocrinol. (2025) 13:949–63. doi: 10.1016/S2213-8587(25)00226-8, PMID: 40961952

[10] Wadden TA Bailey TS Billings LK Davies M Frias JP Koroleva A . Effect of subcutaneous semaglutide vs placebo as an adjunct to intensive behavioral therapy on body weight in adults with overweight or obesity: the STEP 3 randomized clinical trial. JAMA. (2021) 325:1403–13. doi: 10.1001/jama.2021.1831, PMID: 33625476 PMC7905697

[11] Jastreboff AM Aronne LJ Ahmad NN Wharton S Connery L Alves B . Tirzepatide once weekly for the treatment of obesity. N Engl J Med. (2022) 387:205–16. doi: 10.1056/NEJMoa2206038, PMID: 35658024

[12] le Roux CW Steen O Lucas KJ Startseva E Unseld A Hennige AM . Glucagon and GLP-1 receptor dual agonist survodutide for obesity: a randomised, double-blind, placebo-controlled, dose-finding phase 2 trial. Lancet Diabetes Endocrinol. (2024) 12:162–73. doi: 10.1016/S2213-8587(23)00356-X, PMID: 38330987

[13] Jastreboff AM Kaplan LM Frías JP Wu Q Du Y Gurbuz S . Triple-hormone-receptor agonist retatrutide for obesity. A phase 2 trial. N Engl J Med. (2023) 389:514–26. doi: 10.1056/NEJMoa2301972, PMID: 37366315

[14] Garvey WT Blüher M Osorto Contreras CK Davies MJ Winning Lehmann E Pietiläinen KH . Coadministered cagrilintide and semaglutide in adults with overweight or obesity. N Engl J Med. (2025) 393:635–47. doi: 10.1056/NEJMoa2502081, PMID: 40544433

[15] Mehta R Goldenberg R Katselnik D Kuritzky L . Practical guidance on the initiation, titration, and switching of basal insulins: a narrative review for primary care. Ann Med. (2021) 53:998–1009. doi: 10.1080/07853890.2021.1925148, PMID: 34165382 PMC8231382

[16] Eli Lilly and Company . Lilly’s triple agonist, retatrutide, delivered weight loss of up to an average of 71.2 lbs along with substantial relief from osteoarthritis pain in first successful Phase 3 trial. Press release. Dec 11, 2025 (2025). Available online at: https://investor.lilly.com/news-releases/news-release-details/lillys-triple-agonist-retatrutide-delivered-weight-loss-average (Accessed December 30, 2025). Investor Relations, Eli Lilly and Company.

[17] Hayes MR De Jonghe BC Kanoski SE . Role of the glucagon-like-peptide-1 receptor in the control of energy balance. Physiol Behav. (2010) 100:503–10. doi: 10.1016/j.physbeh.2010.02.029, PMID: 20226203 PMC2886183

[18] Alhadeff AL Rupprecht LE Hayes MR . GLP-1 neurons in the nucleus of the solitary tract project directly to the ventral tegmental area and nucleus accumbens to control for food intake. Endocrinology. (2012) 153:647–58. doi: 10.1210/en.2011-1443, PMID: 22128031 PMC3275387

[19] Holst JJ . The physiology of glucagon-like peptide 1. Physiol Rev. (2007) 87:1409–39. doi: 10.1152/physrev.00034.2006, PMID: 17928588

[20] Borner T De Jonghe BC Hayes MR . The antiemetic actions of GIP receptor agonism. Am J Physiol Endocrinol Metab. (2024) 326:E528–36. doi: 10.1152/ajpendo.00330.2023, PMID: 38477667 PMC11194054

[21] Lee SH Park HY Yun JH Do EK . Glucagon in metabolic disease: a mini-review of emerging multi-organ roles beyond glycemic control. Front Endocrinol (Lausanne). (2025) 16:1645041. doi: 10.3389/fendo.2025.1645041, PMID: 40822953 PMC12350081

[22] Eldor R Avraham N Rosenberg O Shpigelman M Golan-Cohen A Cukierman-Yaffe T . Gradual titration of semaglutide results in better treatment adherence and fewer adverse events: A randomized controlled open-label pilot study examining a 16-week flexible titration regimen versus label-recommended 8-week semaglutide titration regimen. Diabetes Care. (2025) 48:1607–11. doi: 10.2337/dc25-0690, PMID: 40673973

[23] Furihata K Mimura H Urva S Oura T Ohwaki K Imaoka T . A phase 1 multiple-ascending dose study of tirzepatide in Japanese participants with type 2 diabetes. Diabetes Obes Metab. (2022) 24:239–46. doi: 10.1111/dom.14572, PMID: 34647404 PMC9299227

[24] Common Terminology Criteria for Adverse Events (CTCAE) v6.0 (MedDRA 28.0) (Bethesda, MD, USA: National Cancer Institute, National Institutes of Health). (2025).

